# Supplementary material for: Tobacco Tax Increases: A Discourse Analysis of the French Print and Web News Media from 2000 to 2020
Source: Int J Environ Res Public Health. 2022 Nov 17;19(22):15152. doi: 10.3390/ijerph192215152 (PMC9691216; doi:10.3390/ijerph192215152)
Supplement: Supplementary file 1 [file ijerph-19-15152-s001.zip › ijerph-1942310-supplementary.pdf]

Table S1 Codes and descriptive write-up for arguments and sub-arguments

	Against
**A)**	**Tax increase is ineffective**
**A1**	Raising tax will have no effect on tobacco use
**A2**	Lower sales does not mean that people have quit smoking
**A3**	Smokers do not quit smoking but switch to vaping
**A4**	Smokers do not quit smoking but switch to cheaper products
**A5**	Smokers anticipate price increases by buying in more (before the increase comes in)
**A6**	Smuggling and cross-border markets make tobacco more accessible to youth
**B)**	**Tax increase causes social and economic damage**
**B1**	There is a loss of revenue for the State
**B2**	There are adverse effects (without giving further details)
**B3**	Tax increases will hurt tobacconists
**B4**	Tax increases will hurt border tobacconists
**B5**	Tax increases will hurt Corsican tobacconists
**B6**	Tax increases will hurt producers
**B7**	Tax increases will hurt industry
**B8**	It is the State's responsibility to support tobacconists in adapting to tax-related changes
**B9**	Tax increases will provoke security problems for tobacconists and delivery services
**B10**	Tax increases risk prompting anti-government votes from tobacconists
**B11**	Tobacco tax is not a reliable source of revenue for funding important social or public health programs
**C)**	**Tax increase disrupts the market**
**C1**	Tax increase fuels the dark market, smuggling, illegal trade
**C2**	It increases the price differences between France and neighboring border countries
**C3**	It increases legal purchases in border countries
**C4**	It increases purchases made online
**C5**	Lockdown in 2020 led to an increase in sales at tobacconists - clients who were purchasing abroad went back to local retailers (they had not quit)
**C6**	Tax increase is not a public health measure but simply a way to pay off State debt
**D)**	**Tax increase has negative impacts for smokers in their day-to-day life**
**D1**	Taxes are regressive and unfair to the poorer
**D2**	Taxes are unfair and punitive toward smokers
**D3**	Taxes are intrusive and constrain smokers' freedoms
**D4**	Taxes are one of the triggers for the 'gilets jaunes' social movement
	In favour
**E)**	**Tax increase is effective method to reduce smoking**
**E1**	Tax increase is an effective measure to reduce smoking / to increase quit attempts
**E2**	It is effective if increases are >10%, continuous, regular, and coupled with other tobacco control measures
**E3**	It is effective if prices of different tobacco products are standardized
**E4**	It is the tobacco industry agreement on prices that mitigates the efficiency of the measures

**E5**	When too weak (<10%), the measure is ineffective for reducing smoking but just a way to enrich the tobacco industry – i.e. it is not a public health measure (the measure has to be ambitious enough)
**E6**	Tax increase does not have the same efficiency across all the population. Tax increases are more or less dissuasive
**F)**	**Tax increase benefits the different parties**
**F1**	Tax increases add to tobacconists' revenue
**F2**	Tax increases add to the tobacco industry's revenue
**F3**	Tax increases will raise general State revenue (dedicated to public health programs)
**G)**	**Some information on negative effects of tax increase is inaccurate or exaggerated**
**G1**	There is no proof of a link between the measure and smuggling or the dark market
**G2**	There is no proof of a link between the measure and increased risk for tobacconists
**G3**	The general population is supportive of the measure
	Alternatives or prerequisites
**H)**	**The taxation strategy alone is not effective to reduce smoking—it needs to work on the back of other measures**
**H1**	Prices should be harmonized within the European Union
**H2**	We need to be tougher in the fight against smuggling and the dark market
**H3**	We should be developing prevention measures for youth instead of increasing taxes
**H4**	We should be advocating lower-risk products as a way to quit smoking instead of increasing taxes
**I)**	**Tax increase will benefit to all if moderate (defends an increase limited at 5%)**

Table S2 Organizational categories of stakeholders and number of citations retrieved

ORGANIZATIONAL CATEGORIES OF STAKEHOLDERS	NUMBER OF CITATIONS RETRIEVED
TOBACCONISTS	2368
POLITICIANS, GOVERNMENT AND REGULATORY BODIES	899
BODIES REPRESENTING HEALTH PROFESSIONALS/PUBLIC HEALTH	853
TOBACCO INDUSTRY	460
ANONYMOUS	282
TOBACCO INDUSTRY AND TOBACCONISTS (SPEAKING AS ONE VOICE)	53
INTERNATIONAL AGENCIES (WHO AND WORLD BANK)	51
CUSTOMSAUTHORITIES	39
POLLING INSTITUTES	38
ACADEMICS (OTHER FIELDS OUTSIDE MEDICINE AND PUBLIC HEALTH)	31
LAW ENFORCEMENT	14
PRODUCERS	10
JOURNALISTS	10
E-CIGARETTE RETAILERS	3
JUSTICE	3

Table S3 Number of arguments per year (from 2000 to 2010) * See Table S1 for the names for each argument .

Category	Years										
themes and arguments	2000	2001	2002	2003	2004	2005	2006	2007	2008	2009	2010
Against	11	16	189	1006	305	97	77	153	49	270	214
A	2	1	34	135	79	16	11	34	8	45	41
A1	2	1	22	77	36	2	5	15	6	34	28
A2			2	15	17	6		5			2
A3											
A4			4	14	17	7	6	6	2	9	10
A5			4	19	7			8		2	1
A6			2	10	2	1					
B	8	5	64	503	117	42	53	54	16	63	30
B1			23	32	12	3		1	1	3	5
B2			2	5		1					
B3	7	2	17	219	60	16	36	41	11	43	12
B4			12	83	20	11	11	10	2	8	9
B5											
B6			3	4						6	
B7		2	4	11	6	1	1		2	1	
B8											
B9	1	1	3	113	17	4	1	2		2	4
B10				33	2	6	4				
B11				3							
C	1	7	88	338	104	38	13	63	23	120	93
C1	1	3	66	257	61	26	6	32	6	48	45
C2				4	8			1	1	3	3
C3			14	19	26	8	7	29	16	49	31
C4				3	3	4				20	7
C5											
C6		4	8	55	6			1			7
D		2	3	30	5	1		2	1	3	10
D1			1	29	5	1		2	1	3	10
D2		2	2								
D3				1							
D4											
In favor	24	18	51	357	149	21	38	33	32	93	172
E	16	13	23	242	130	13	29	23	20	86	155
E1	9	3	13	182	112	9	9	14	2	26	13
E2	7	10	5	54	13	2	17	5	4	45	91
E3			5		1				14	1	2
E4											
E5				1	1	1	3	4		12	47
E6				5	3	1				2	2

F	5	5	23	65	19	7	7	10	12	7	16
F1			1	12	17	6	6	1	4	3	10
F2	4	5	16	18	2	1	1	9	8	4	6
F3	1		6	35							
G	3		5	50		1	2				1
G1	3		5	30		1					1
G2				16							
G3				4			2				
Alternatives		1		6	1			1	4	41	40
H				6	1			1	3	2	
H1				6	1			1		1	
H2									3	1	
H3											
H4											
I		1							1	39	40

Table S4 Number of arguments per year (from 2011 to 2020) *See Table S1 for the names for each argument

**Category themes and arguments**	Years									
	2011	2012	2013	2014	2015	2016	2017	2018	2019	2020
Against	175	483	547	281	70	171	536	232	216	200
A	40	102	112	74	12	35	118	49	37	44
A1	28	65	75	45	12	31	103	24	20	30
A2	9	14	19	10		1	3	13	9	3
A3		1	6	9			6	7	7	4
A4	2	20	12	10		3	6	2		7
A5	1	1						3	1	
A6		1								
B	35	112	138	88	25	57	123	89	76	29
B1	4	30	8	13	1	8	9	3		2
B2		2								1
B3	16	55	74	55	19	31	91	48	36	9
B4	9	11	9	2	3	8	4	3	1	8
B5									6	
B6						4				
B7		2	20	12		6	1	1	3	
B8								21	20	9
B9	6	12	27	6	2		18	13	10	
B10										
B11										
C	88	251	252	110	32	69	258	92	98	120
C1	55	114	146	72	19	38	171	65	58	51
C2	1	21	5	12	8	3	15		5	4
C3	28	93	81	19	3	28	53	20	29	22
C4	3	19	11	7			12		2	1

C5					1					42
C6	1	4	9		1		7	7	4	
D	6	8	4	6	1	9	36	2	5	7
D1	6	8	4	2	1	9	31	2	1	5
D2				4			5		2	2
D3										
D4									2	
In favor	78	181	246	121	71	190	280	180	169	72
E	71	166	231	114	64	187	261	173	150	66
E1	12	48	103	57	24	51	114	112	76	52
E2	25	77	64	32	24	64	112	40	61	11
E3	3	12	5	3	2	49	10	3	7	2
E4			11							
E5	31	27	48	22	11	22	16	11	4	
E6		2			3	1	9	7	2	1
F	2	13	12	7	6	2	9	7	18	2
F1	2	11	8	5	4		6	3	9	1
F2		1	4	2	2	1			2	
F3		1				1	3	4	7	1
G	5	2	3		1	1	10		1	4
G1	5	2	3		1	1	4		1	2
G2										
G3							6			2
Alternatives	6	12	44	16	3	13	65	4	6	21
H		2	3	13	3	12	64	4	6	21
H1		2	1	11	3	7	33		6	13
H2			2	2		5	14			1
H3							17	2		3
H4								2		4
I	6	10	41	3		1	1			

Table S5 Occurrences of citations per years for the most frequent speakers

**Years**	**Most frequent speakers**			
	**Tobacconists**	**Politicians Government Regulatory bodies**	**Tobacco industry**	**Bodies representing health professionals/ public health**
2000	4	1	1	2
2001	3	4	1	9
2002	60	21	15	7
2003	403	160	65	87
2004	128	12	56	35
2005	35	3	4	7

2006	27	5	2	13
2007	100	5	2	7
2008	16	14	9	2
2009	9	69	16	47
2010	84	20	19	95
2011	68	6	5	48
2012	237	79	43	78
2013	288	97	43	98
2014	132	62	13	44
2015	33	46	6	18
2016	89	103	20	32
2017	291	156	60	86
2018	93	17	22	57
2019	90	11	25	46
2020	93	8	33	35
**Total**	2368	899	490	853
